# A Dimensionality Reduction-Based Multi-Step Clustering Method for Robust Vessel Trajectory Analysis

**DOI:** 10.3390/s17081792

**Published:** 2017-08-04

**Authors:** Huanhuan Li, Jingxian Liu, Ryan Wen Liu, Naixue Xiong, Kefeng Wu, Tai-hoon Kim

**Affiliations:** 1 Hubei Key Laboratory of Inland Shipping Technology, School of Navigation, Wuhan University of Technology, Wuhan 430063, China; huanhuan.li@whut.edu.cn (H.L.); ljxteacher@sohu.com (J.L.); 2 National Engineering Research Center for Water Transport Safety, Wuhan 430063, China; 3 School of Mathematics and Computer Science, Northeastern State University, Tahlequah, OK 74464, USA; 4 School of Automation, Huazhong University of Science and Technology, Wuhan 430074, China; m201472334@hust.edu.cn; 5 Department of Convergence Security, Sungshin Women’s University, 249-1 Dongseon-dong 3-ga, Seoul 136-742, Korea; taihoonn@empal.com

**Keywords:** vessel trajectory clustering, the improved center clustering algorithm, DTW, PCA, spectral clustering

## Abstract

The Shipboard Automatic Identification System (AIS) is crucial for navigation safety and maritime surveillance, data mining and pattern analysis of AIS information have attracted considerable attention in terms of both basic research and practical applications. Clustering of spatio-temporal AIS trajectories can be used to identify abnormal patterns and mine customary route data for transportation safety. Thus, the capacities of navigation safety and maritime traffic monitoring could be enhanced correspondingly. However, trajectory clustering is often sensitive to undesirable outliers and is essentially more complex compared with traditional point clustering. To overcome this limitation, a multi-step trajectory clustering method is proposed in this paper for robust AIS trajectory clustering. In particular, the Dynamic Time Warping (DTW), a similarity measurement method, is introduced in the first step to measure the distances between different trajectories. The calculated distances, inversely proportional to the similarities, constitute a distance matrix in the second step. Furthermore, as a widely-used dimensional reduction method, Principal Component Analysis (PCA) is exploited to decompose the obtained distance matrix. In particular, the top *k* principal components with above 95% accumulative contribution rate are extracted by PCA, and the number of the centers *k* is chosen. The *k* centers are found by the improved center automatically selection algorithm. In the last step, the improved center clustering algorithm with *k* clusters is implemented on the distance matrix to achieve the final AIS trajectory clustering results. In order to improve the accuracy of the proposed multi-step clustering algorithm, an automatic algorithm for choosing the *k* clusters is developed according to the similarity distance. Numerous experiments on realistic AIS trajectory datasets in the bridge area waterway and Mississippi River have been implemented to compare our proposed method with traditional spectral clustering and fast affinity propagation clustering. Experimental results have illustrated its superior performance in terms of quantitative and qualitative evaluations.

## 1. Introduction

**Figure 1:**
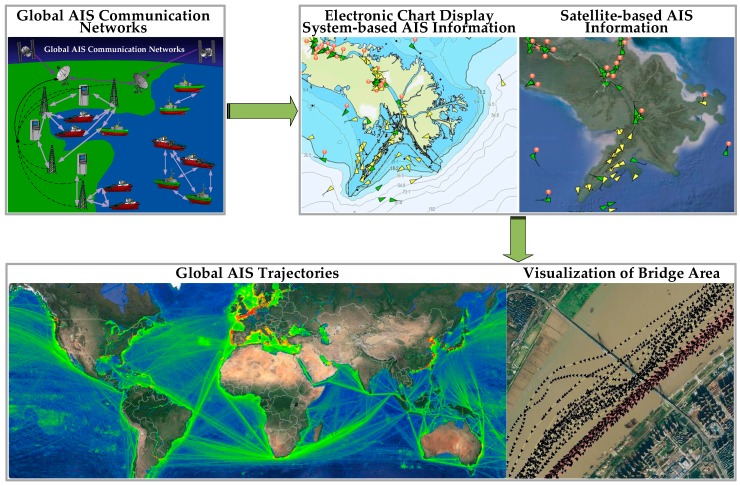
Visualization of different AIS information [8,9].

Automatic Identification System (AIS) is used to identify and locate vessels by electronically exchanging data with other nearby ships and Vessel Traffic Services (VTS) stations. To enhance the maritime traffic safety, it is important to extract navigational characteristics and rules through analyzing the maritime traffic patterns [1,2]. With the rapid development of shipboard AIS in practice, data mining methods have been widely used to explore the maritime traffic patterns and maritime situational awareness activities based on massive AIS datasets [3]. In particular, AIS data can be effectively used to infer different levels of contextual information from the characteristics of ports and off-shore platforms to spatial and temporal distributions of routes [4,5]. Global AIS networks as a unity of satellite AIS and terrestrial AIS are consisted of AIS communication system all over the world, which include a wealth of AIS trajectories. The AIS visualization technology is helpful to the dynamic regulation and real-time monitoring of vessels, then realizing the seamless global tracking of vessels throughout the world [6,7]. Visualization of different AIS information is shown in Figure 1.

**Figure 2:**
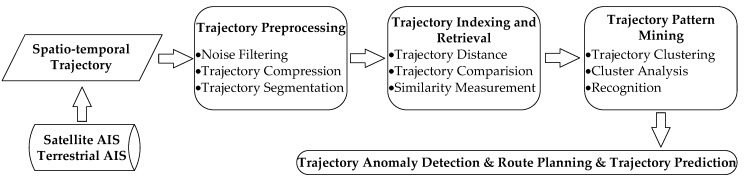
The main modules of AIS trajectory data mining.

The original AIS data contains massive noise and redundant information. Supervisors need to get effective and real-time AIS information to manage and regulate vessels. Captains also need to know the movement status of the surrounding vessels to operate their vessels. Thus, how to extract reliable and useful AIS trajectory features has become the critical problem [10,11]. Ways of extracting real-time and accurate vessel information have also become a research focus [12]. AIS trajectory data mining mainly contains five research contents: trajectory data collection, trajectory preprocessing, trajectory indexing and retrieval, trajectory pattern mining and anomaly detection. The main theories of trajectory data mining have been reviewed in detail [13]. The main research modules of trajectory data mining are presented in Figure 2. Trajectory preprocessing is necessary to verify the accuracy of data. Trajectory indexing and retrieval can compare the difference and measure the distance between trajectories. Trajectory pattern mining can aid to the trajectory analysis, and also will further apply to detect abnormal trajectory, plan the route and predict trajectory in the real world [14].

AIS is a reporting system for ships that can broadcast accuracy locations and further ensure navigation safety and was originally conceived for collision avoidance [15]. At present, the domestic and foreign research on the application of AIS data mainly consist of five aspects. The most common applications are collision avoidance and anomaly detection. The other three applications are vessel behavior identification, the extraction of knowledge and the vessel tracking and prediction. Statheros et al. [16] have reviewed the reasons and research methods of ship collision avoidance clearly, and analyzed the human navigation ability and the different ship collision avoidance mathematical models. Mou et al. [17] proposed a linear regression model based on AIS data to study the traffic characteristics and avoid collisions in busy waterways, and eventually constructed a dynamic model to assess the risk. Bomberger et al. [18] and Rhodes et al. [19] developed refined models to learn the normal patterns and evaluate the behavior patterns, then predict the vessel location based on the artificial neural network method. Ristic et al. [20] analyzed historical AIS data to mine the motion patterns, and proposed an algorithm to detect anomalies based on the adaptive kernel density estimation, eventually to predict the vessel motion based on the Gaussian sum tracking filter. Mascaro et al. [21] proposed dynamic and static Bayesian network models for anomaly detection, which is based on the normal behavior and anomalous behavior information extracted from the typical AIS data. Mazzarella et al. [22] proposed an automatic extraction method to obtain real knowledge from historical AIS data and assess the behavior of fishing vessels, and thus automatically discover fishing areas. The non-spatial attributes speed and direction were taken into the clustering algorithm [23], and the DBSCANSD algorithm was proposed to extract the normal trajectories and discern the abnormal patterns, and eventually to mine the traffic patterns and monitor maritime traffic. Vespe et al. [24] introduced a density map to identify fishing activities and investigated the mapping fishing activities based on the use of AIS data to track vessels. The architecture and theory of the three AIS receivers in space were introduced clearly by Skauen [25], and a method of tracking and quantifying the tracking capability was also proposed. Pallotta et al. [26] explored the traffic routes in TREAD and proposed a useful prediction method based on the popular Ornstein-Uhlenbeck stochastic processes to locate vessels and predict the accuracy of vessel locations, thus further ensuring maritime security. AIS data contains a vast amount of information to aid navigation safety, vessel traffic services and maritime domain awareness [27]. Therefore, mining of AIS data is crucial to exploit the full potential of different applications. Certainly, there are many risks of spoofing and creating counterfeit messages in AIS to launch an attack. The theory of AIS systems and various AIS spoofing methods were described in detail [28], and the unique security evaluation of AIS was introduced. The novel software AISTX was proposed to further ensure and improve the security.

In this paper, we mainly extract the characteristics of AIS data by trajectory clustering, then find customary routes and discern the abnormal trajectories. The clustering analysis of vessel trajectories can provide a theoretical basis for the design of route planning and management system. It is also helpful to strengthen the dynamic monitoring of ships and improve the efficiency of maritime supervision.

In conclusion, the main contributions of this paper can mainly be summarized by the following three aspects: (1) the dimension reduction method is introduced to determine the number of cluster centers; (2) the improved center clustering algorithm can automatically select the centers based on the distance between trajectories; (3) fusion of Dynamic Time Warping (DTW), Principal Component Analysis (PCA), and the proposed center clustering algorithm can better deal with the trajectory clustering. The good performance of our proposed AIS trajectory clustering method was confirmed by numerous experiments.

The remainder of this paper is organized as follows: in Section 2, we provide a literature review of trajectory clustering methods and briefly describe the traditional clustering methods. In Section 3, the multi-step clustering method to analyze the spatio-temporal AIS trajectories is proposed and described clearly. In Section 4, numerous experiments on realistic AIS trajectory datasets have illustrated the necessity and effectiveness of the proposed method in practical applications. Finally, we conclude this paper by summarizing our contributions and pointing out some future work directions in Section 5.

## 2. Literature Review of Clustering Methods

Clustering is one of the important research methods of data mining, which is conducive to obtaining pattern information about vessels. The clustering process is known as an unsupervised learning method, for which no prior knowledge about the data is needed. It groups data with different clustering algorithms [29,30,31]. Recently, a large number of clustering algorithms have been proposed, for example k-means [32], spectral clustering [33], Density-Based Spatial Clustering of Applications with Noise (DBSCAN) [34], Balanced Iterative Reducing and Clustering using Hierarchies (BIRCH) [35] and so on. In recent years, clustering methods have attracted increasing attention of researchers in the fields of data mining and pattern recognition. Up to now, the current clustering methods could be roughly divided into five categories, i.e., partitioning methods [36,37] (e.g., K-means, K-mediods), hierarchical methods (e.g., BIRCH), density-based methods (e.g., DBSCAN [38]), grid-based methods [39] (e.g., STING [40]), and model-based clustering methods [41].

Specifically, partition-based clustering techniques [42] firstly create an initial partition, then positioning technology is repeatedly adopted to optimize the clustering results. The optimization criterion is based on the variation of objects in different partitions. The optimal partition is not achieved until the partition result satisfies the minimum objective function value. The partition criterion is that the similarity of any two objects in the same cluster be as large as possible, and the similarity of the data objects of different classes be as small as possible.

Hierarchy-based clustering algorithm [43] can be further divided into three kinds, bottom-up algorithms or condensation algorithms [44] (e.g., CLIQUE, MAFIA and ENCLUS), top-down algorithms or decomposition algorithms [45] (e.g., PROCLUS and ORCLUS) and compound algorithms (e.g., BIRCH and CURE). The traditional algorithms BIRCH and CURE are not applicable due to the large amount of computation.

The essence of density-based clustering algorithms is that the high density areas are separated by low density areas. The density algorithms are different from each other because of the different definitions of high and low density areas. Typical algorithms are DBSCAN, OPTICS [46], CLIQUE and so on.

Grid-based clustering algorithms will form a grid structure by quantifying object space to a finite number of cells. Then all of the clustering operations are performed on the grid structure (i.e., the quantization space). Grid-based clustering algorithms mainly include Statistical Information Grid-based method (STING), Clustering with Wavelets (WaveCluster) [47], Clustering In QUEst (CLIQUE) and so on. Grid-based clustering algorithms are suitable for clustering in large datasets, however they are sensitive to the input parameters, and also are difficult to find an effective method for setting the parameters in theory.

The basic idea of model-based clustering algorithms [48] is to find the best match between the given data and a mathematical model. Model-based clustering algorithms are used to handle the dataset containing the noise data and isolated points. They can reflect the distribution of data points by constructing the effective density function and automatically obtain the number of clusters. Which makes them a robust clustering method. Model-based clustering methods mainly include statistics-based clustering methods [49].

The primary purpose of this paper is to discover the movement patterns and discern anomalous trajectories, then establish the traffic rules and maintain maritime security. The traffic flow patterns detected by the clustering method from the original trajectories data have provided a solid foundation for further research on trajectory visualization [50,51]. This can be used for mining customary routes and establishing safe routes [52]. It is well known that trajectory data is essentially different from traditional point data. The commonly used point clustering algorithms cannot be directly adopted to handle trajectory clustering [53,54,55]. There are many basic questions that need to be considered for trajectory clustering. For instance, how to consider the whole trajectory length, how to extract the useful traffic flow information from AIS trajectories, how to choose the appropriate points in AIS trajectories, how to directly measure the similarity between different trajectories and so on [56].

A trajectory is composed of a series of points, and has a linear or nonlinear structure with respect to time. The different trajectories of moving objects have their own unique attributes, so trajectory clustering analysis is more complex than traditional point clustering. There are generally two ways to study trajectory clustering, one is to regard the trajectory as a whole, and the other is to partition the trajectory into a set of line segments.

The trajectory can be taken as a whole, and scholars have reported some research findings in this area. Gaffney et al. [57] proposed a trajectory clustering method based on the probabilistic model and the mixed regression model. The maximum likelihood principle and the expectation-maximization algorithm were applied in trajectory clustering. Nanni et al. [58] proposed dynamic trajectory clustering based on density, where the similarity between trajectories was measured with the average distance between the nodes in trajectories. Gaffney et al. [59] developed a probabilistic clustering technique and a regression mixture model to describe the geographic location and track trajectories. Pan et al. [60] proposed a new trajectory clustering algorithm called TRASAD based on sampling and DBSCAN to cluster the whole trajectory, and the entropy theory and the heuristic algorithm were chosen to select the parameters. However, how to choose the fit similarity measurement method to measure the distance between trajectories is the critical problem in the whole research method. Moreover, how to select the number of cluster centers and the clustering algorithm are also problems.

The partition method can divide the trajectory into similar sub-trajectories based on the geometric features and structural similarity. Then the sub-trajectories can be clustered by traditional clustering methods. Lee et al. [61] proposed a partition-and-group framework based on the minimum description length, then developed the TRACLUS algorithm based on DBSCAN. In this method, the trajectory is divided into a set of sub-trajectories, and TRACLUS is used to cluster the sub-trajectories. Lee et al. [62] proposed a novel partition-and-detect framework and two steps trajectory partitioning strategy to discern trajectory outlier. The algorithm firstly divided the trajectory into sub-trajectories, and then detected the outlying sub-trajectories. Lee et al. [63] improved the TRACLUS and constructed the feature generation framework TraClass, which can generate the hierarchical features based on the trajectories segmentation. Yu et al. [64] proposed a clustering algorithm CTraStream based on density to cluster the sub-trajectory data stream and update the online trajectories. However, the partition method also have some problems, for example how to determine the length of sub-trajectories, how to measure the distance between the sub-trajectories and so on.

## 3. Proposed Method

### 3.1. The Proposed Multi-Step Clustering Algorithm

In order to preserve the full shape and structure features of the trajectories, every trajectory is taken as a whole in this paper. To improve the accuracy of distance calculation between different trajectories, DTW has been introduced as a trajectory similarity measurement method [65]. When the distance matrix is received, the clustering algorithm based on distance matrix should be chosen. A novel method for determining the number of cluster centers is introduced, which is based on the dimensional reduction method PCA [66]. PCA was then introduced to reduce the dimension of distance matrix and calculate the top *k* principal components. The clustering number *k* is determined by the accumulative contribution rate of the top *k* principal components. At last, the improved center clustering algorithm was implemented to perform AIS trajectory clustering with the known cluster number *k*. The proposed multi-step framework could significantly improve the clustering accuracy of AIS trajectories.

A trajectory is composed of a large number of points, and the trajectory similarity measurement method can measure the distance between trajectories. Many methods have been proposed to measure the distance between different points [67,68]. Previous works of different scholars have proposed many popular distance measurement methods. For instance, simple Euclidean distance, Hausdorff distance, Hidden Markov Model (HMM), DTW, Longest Common Subsequence (LCSS) and so on. The length of all trajectories must be equal in Euclidean distance. Hausdorff distance has no demand for the length of trajectory, however it is time-consuming. HMM distance gives each trajectory a statistical model, however it has high time complexity. The scholars have verified both Hausdorff and HMM have poor performance [69]. Compared with location similarity, LCSS pays more attention to shape similarity and has high time cost. DTW is easier to find the shape similarity of the trajectory, and is warping the route from feature to feature. Therefore, DTW is chosen as the similarity measurement method in this work to calculate the distances between trajectories.

DTW [70] is an algorithm for measuring similarity between two time series, and the essence is warping the route from feature to feature. DTW is a kind of nonlinear programming techniques based on the time programming and distance test. It can be used to calculate the similarity between two time series, and eventually find the shortest distance [71,72,73]. DTW can find an optimal path with a minimum cost based on dynamic programming.

As a classical data dimension reduction algorithm, PCA can not only reduce the dimension of high dimensional data, but also remove the noise and find the hidden pattern in the data. The number of clusters can be obtained by selecting the top *k* principal components whose accumulative contribution rate is greater than 95%. From the point of view of image recognition, the top *k* principal components whose accumulative contribution rate is more than 95% contain most of the image information. The top *k* principal components of the distance matrix are orthogonal and linear independent, then the similarity between the top *k* principal components is very low.

Each trajectory is regarded as a whole, then the trajectory clustering problem is transformed into an abstract point clustering problem. The trajectory is composed of many points and other information, and can visualize the motion of the vessels. The multi-step clustering algorithm is proposed to solve the trajectory clustering problem, eventually to ensure the safety and security of maritime traffic, detect anomaly behavior and avoid collision. Our proposed multi-step clustering method can not only reduce the data dimensionality, but also provide a better clustering effect without increasing the time complexity.

**Figure 3:**
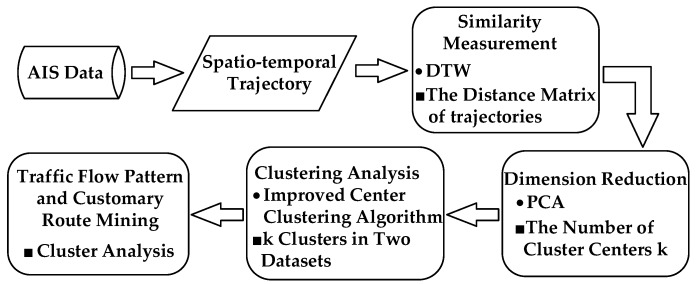
The flow chart of the multi-step clustering algorithm.

The proposed multi-step clustering algorithm can receive the AIS trajectories clustering results to analysis the mobility patterns and discover the characteristics of moving vessels. The technical flow chart of the fusion algorithm is illustrated in Figure 3.

### 3.2. The Improved Center Clustering Algorithm

K-means clustering is a kind of partition-based clustering methods, which needs to set *k* points as the clustering centers. This algorithm randomly selects *k* points as the cluster centers, then the remaining points are assigned into the nearest cluster according to the distance between the remaining points and the center of each cluster [74]. The K-means algorithm takes the mean value of the points in one cluster as a center, which may not be the actual data point.

Spectral clustering is based on the spectral graph partition theory; its essence is transforming the clustering problem of sample space into an optimal graph partition problem. Spectral clustering can divide the graph into several subgraphs, which have no intersection between each other. Spectral clustering can identify the sample space with arbitrary shape and converge to the global optimal solution. However, it is not suitable for the datasets with many clusters. The basic idea of spectral clustering is to classify the feature vectors, which is received by the feature decomposition.

The improved center clustering algorithm is proposed herein to cluster AIS trajectories, and it is based on the essence of K-means algorithm and spectral clustering. It can automatically select the cluster centers. The algorithm combines with PCA to define the number of clusters based on the top *k* principal components and the accumulative contribution rate. Then cluster analysis is carried out to mine the hidden patterns and characteristics according to the distance matrix. The principal components of distance matrix denote the main features, and it’s effective to extract the trajectory information and remove low correlation information.

Assume that *m* denotes the number of the trajectories in database, then there are $C_{m}^{2}$ distances, all the distances are sorted in descending order. $d_{i+1}\;,\;i=1\;,\;\cdots ,\;C_{m}^{2}$ represents the *i^th^* distance value. $\xi_{i}=|d_{i}-d_{i+1}|,\;i=1\;,\;\cdots ,\;C_{m}^{2}$ is the absolute value of difference between two adjacent distances. The threshold value is $\xi =(\sum_{i=1}^{C_{m}^{2}}d_{i})/C_{m}^{2}$. The improved center clustering algorithm steps describe as follows.

**Algorithm 1. The Improved Center Clustering Algorithm**1: **Input**: *k* //the number of clusters

2:     ξ //the threshold value
3:      $l_{i},i=1,\ldots ,m$ //the label of every trajectory4: **Output**: the cluster results5: /*Abnormal trajectories detection*/6:  (a) **IF**
$\xi_{i}\geq \xi$7:    **then**
$l_{i}=0$, the trajectory perhaps is abnormal.8:     /*Identifying abnormal trajectories*/9:     **if**
*SOG* = 0, or $SOG>2V_{average}$, or $COG[(t_{i}-t_{i+1})>2\min]>30^{\circ }$10:     // $V_{average}=(\sum_{i=1}^{C_{m}^{2}}V_{i})/C_{m}^{2}$, $V_{i},i=1,\;\ldots ,\;m$ denotes the speed of every vessel.
11:     // $t_{i}-t_{i+1}$ means the time difference.12:      **then** delete the abnormal trajectories.13:     **else**14:     modify $l_{i}=1$.15:     **end**16:  (b) **ELSE**17:    **then**
$l_{i}=1$, normal trajectories, enter the next step.18:    **end**19: /*The clustering center automatic selection algorithm*/20: **for**
*k = 2* to *m*
**do**21:  (a) **IF**
*k = 2*22:    **then** the two trajectories corresponding to the maximum distance are taken as the clustering centers.23:   **end**24:  (b) **ELSE**25:    find the top *k* maximum distance26:    **if** the top *k* maximum distance are the distance among *k* trajectories,27:      **then** the *k* trajectories are taken as the clustering centers.28:    end29:      **if** the top *k* distance are formed by the [*k* + 1, 2*k*] trajectories,30:      **then** choose the *k* trajectories which are repeated most often as the cluster centers.31:     **end**32:   **end**33: /*Cluster analysis and trajectory pattern mining*/34: (a) The trajectories are grouped into *k* clusters35:   // Trajectory clustering according to the known k centers.36: (b) Cluster analysis37:   // Find the custom routes and make safety routes.

## 4. Performance Analysis

To verify the accuracy and efficiency of the proposed method, numerical experiments were performed based on realistic AIS trajectory datasets in the Bridge Area Waterway and Mississippi River. The Bridge Area Waterway is a research focus of inland waterways. Once a vessel collision happens in the bridge area, it will not only have a serious impact on inland waterway traffic, but also potentially lead to serious consequences such as bridge collapse. On the other hand, the complex and changeable waters of vessel traffic flow are also the research focus. Therefore, AIS trajectory clustering and visualization become the research focus, which can discover customary routes and detect abnormal behavior to reduce accident rates and prevent accidents.

### 4.1. Experimental Setup

In this paper, the proposed multi-step clustering algorithm is compared with spectral clustering and fast affinity propagation clustering with specific cluster centers on realistic AIS trajectory datasets from the bridge area waterway on the Yangtze River and the Mississippi River. All numerical experiments were performed using 64-bit Windows 10 on a 2.60 GHz Intel Core i7-5600U CPU equipped with 8 GB memory. We implemented multiple clustering methods using MATLAB R2016a. The experimental data was collected from the AIS base station in Wuhan section in the Yangtze River and the Mississippi River. The bridge area waterway datasets include AIS trajectory data of 187 vessels with 29,015 points. The Mississippi River datasets include 106 AIS trajectories, and 2442 points altogether.

The experimental procedure was as follows:

- Step 1: Trajectory data acquisition and preprocessing.
- Step 2: The similarity measurement of AIS trajectories by DTW.
- Step 3: Dimension reduction processing and the selection of cluster number.
- Step 4: The selection of the clustering centers based on the improved center clustering algorithm.
- Step 5: Clustering analysis based on the improved center clustering algorithm is carried out to receive the best cluster results.
- Step 6: The clustering performance comparison and analysis of different algorithms.

**Figure 4:**
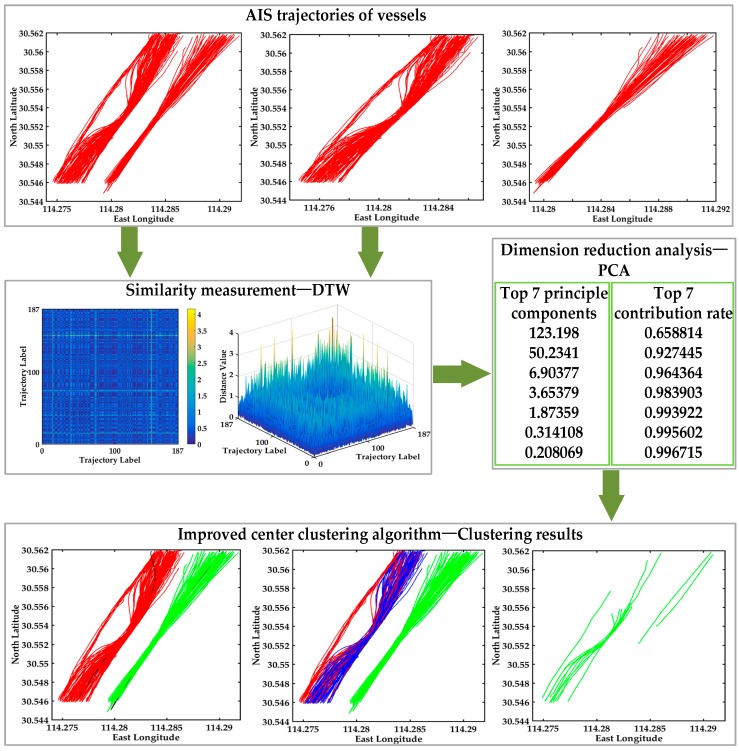
The experimental flow chart of the multi-step clustering algorithm.

The experimental flowchart of the multi-step clustering algorithm (without data preprocessing) is shown in Figure 4. The original AIS trajectories of vessels are displayed, and subsequently the similarity measurement method DTW is introduced to calculate the distance. The 2D and 3D image are displayed in Figure 4, which is conductive to visualize the abnormal trajectories. Then PCA is used to decompose the distance matrix, and the top *k* principal components whose accumulative contribution rate over 95% are received. Then the number of clusters *k* is determined. It can clearly see the accumulative contribution rate of top seven principal components, and the accumulative contribution rate 95% is between the top two accumulative contribution rates and the top three accumulative contribution rates. In order to ensure the validity of the experiments, the clustering results of *k* = 2 and *k* = 3 are compared in Figure 4. Finally, the improved center clustering algorithm is used to define *k* centers and cluster the trajectories to analyze customary routes and detect abnormal trajectories. The clustering results of the improved center clustering algorithm are clearly shown in Figure 4, the clustering performance of *k* = 2 is better than *k* = 3, however the accumulative contribution rate of *k* = 2 is less than 95%. The main reason is that the raw trajectory data has not been pretreated, and all the abnormal trajectories in Figure 4 are incomplete trajectory data, therefore data preprocessing is necessary for trajectory clustering. The following experiments are implemented on the basis of data preprocessing to verify the effectiveness of our proposed multi-step clustering algorithm.

### 4.2. Clustering Analysis of AIS Trajectories in the Bridge Area Waterway

#### 4.2.1. Visualization of the Distance Matrix

The hydrodynamic interactions between bridges and vessels make bridge area waterways a high risk area. Our research can realize the visualization of AIS trajectories in bridge area waterways, and further can deal with abnormal trajectory and discover customary routes by clustering analysis. Then find the vessel traffic flow model and detect abnormal trajectories.

Data cleansing is the basic step of trajectory visualization, and it can delete erroneous data and repair incomplete data. The incomplete and invalid trajectory data are deleted by judging the original abnormal trajectories according to the trajectory acquisition time and time interval. The original abnormal trajectories have many different types. For instance a trajectory may have only one point or two points, or the trajectory was only recorded during the first half of the time, or lacks coordinate data and so on. In the end 161 trajectories of different vessels in the bridge area waterway were preserved after data cleansing.

**Figure 5:**
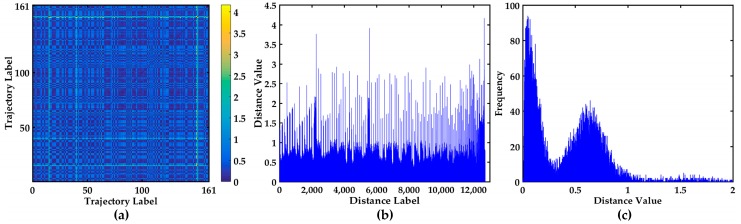
Visualization of the 161 × 161 distance matrix. (**a**) 2D image visualization of distance matrix; (**b**) The bar chart of all the different values in 161 × 161 distance matrix; (**c**) The statistical histogram of all the non-repetitive distances in 161 × 161 distance matrix.

DTW is implemented to calculate the distance between trajectories. The 161 × 161 distance matrix is received, and the visual display of the 161 × 161 distance matrix is shown in Figure 5. The 2D image visualization of distance matrix is shown in Figure 5a. The 161 × 161 distance matrix is symmetric, and half of the elements are repeated. To clearly see the distance distribution between all the trajectories, the bar chart and the statistical histogram of all the non-repetitive distances in 161 × 161 distance matrix are also shown in Figure 5b,c. The bar chart shows the distribution of all the values and provides the visualization of distance matrix, which is conductive to analysis the abnormal trajectories. The trajectories corresponding to these peaks that are much larger than others may be abnormal trajectories. The statistical histogram is conductive to finding the distribution of all the distances. The distances are mainly distributed in [0, 0.4] and [0.4, 1], which indicates that the trajectories are relatively centralized.

#### 4.2.2. Visualization of the Clustering Number

PCA is conducted to reduce the dimensions of the distance matric and decompose the distance matrix. Then *k* is chosen according to the accumulative contribution rate. The top 10 eigenvalues of the distance matrix and the corresponding accumulative contribution rate are listed in Table 1. It’s obvious that the accumulative contribution rate of the top two eigenvalues is 96.67%, which is more than 95%. Therefore the number of cluster is set to be *k = 2* in the proposed multi-step clustering algorithm, and *k = 2* is also taken as the number of clusters in the improved center clustering algorithm. Certainly the experiments will compare the clustering performance when *k = 2* and *k = 3* to verify the accuracy of the number of clusters.

#### 4.2.3. Visualization of Clustering Results in the Bridge Area Waterway

Experiments were carried out to compare and analyze the performance of three algorithms: the proposed multi-step clustering method, spectral clustering and affinity propagation clustering.

In this experiment, the number of the clusters is set to be 2 according to PCA, and the centers are selected from the distance matrix. Through sorting the distance between all the trajectories, there are $C_{161}^{2}=12,880$ distances between 161 trajectories. $d_{i},\;i=1,\;\cdots ,\;12,880$ represents the *i^th^* distance value. $\xi_{i}=|d_{i}-d_{i+1}|\;,\;i=1,\;\cdots ,\;12,880$, which is the absolute value of difference between two adjacent distances. ξ denotes the threshold value. AIS trajectory in the inland river is more regular and smoother than the open seas. The threshold is the average value of all the distances, herein the threshold value is set to $\xi =(\sum_{i=1}^{12880}d_{i})/12880=0.4235$. $\xi_{i}>0.4235$ expresses that the corresponding trajectories may be abnormal trajectories. Then the corresponding trajectories are further confirmed whether they are abnormal trajectories according to Course Over Ground (COG) or Speed Over Ground (SOG).

Through sorting the distance in descending order between all the trajectories, we can clearly see the maximum and minimum distance. The top 10 distances between trajectories are shown in Table 2, and the minimum distance is 0.0077. It can be seen that the values $\xi_{i}<\xi$ from Table 2, however the maximum difference may not have occurred yet.

Then all the distances between trajectories are sorted in descending order, and the top k maximum distances may be anomalous trajectories. To further identify the abnormal trajectories, the differences between distances are applied to find the hidden abnormal trajectories.

**Figure 6:**
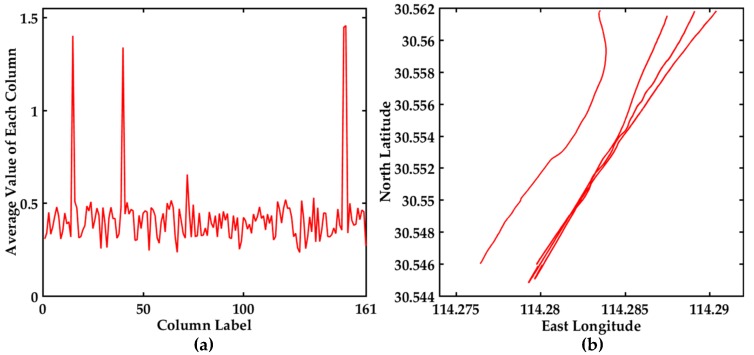
Visualization of the average value and possible abnormal trajectories among 161 trajectories, (**a**) The display of the average value of each column in the distance matrix of 161 trajectories; (**b**) The trajectories corresponding to the top three average values.

The commonly used average value method is to visualize the average value of each column in the distance matrix of 161 trajectories. The top *k* maximum trajectories may be the abnormal trajectory. The visualization display is shown in Figure 6. The average value of each column in the distance matrix of 161 trajectories is shown in Figure 6a, and there are three values obviously larger than other values. The trajectories corresponding to the top three average values are shown in Figure 6b, which is conductive to flag abnormalities. This method can directly discern the obvious anomalous trajectories, but it is difficult for it to find hidden abnormal trajectories.

**Figure 7:**
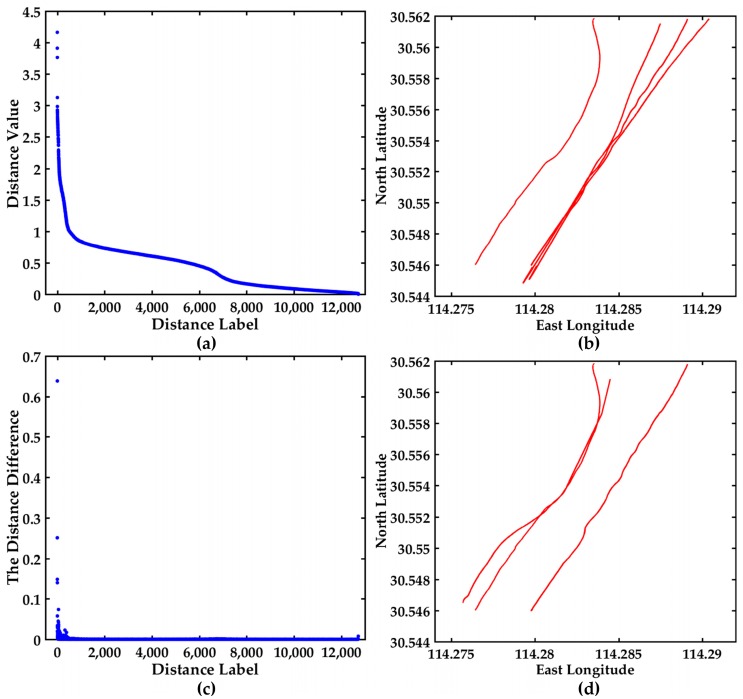
Visualization of the distance values between 161 trajectories and the corresponding trajectories, (**a**) The descending order map of distance values; (**b**) The trajectories display corresponding to the top three distances; (**c**) The distance difference display between two adjacent distance values in (a); (**d**) The trajectories display corresponding to the maximum distance difference.

To find the hidden abnormal trajectories, the visual display of all the distances among the 161 trajectories and the corresponding trajectories in the bridge area are shown in Figure 7. The descending order map of all the distances is shown in Figure 7a, which can clearly see the variation trend of the distances. The maximum distance is 4.160986 in Figure 7a. The distance difference between two adjacent distance values is shown in Figure 7c. The difference $\xi_{i}=0.638066$ between the third distance and the fourth distance is the largest. It can be seen from Table 2 and Figure 7 that $\max\xi_{i}>\xi$, and the top *k* trajectories corresponding to the maximum distance perhaps the anomaly trajectories. Then it is further confirmed if the top *k* trajectories are abnormal trajectories based on COG and SOG. The COG and SOG don’t satisfy the abnormal conditions, so the top three trajectories are not anomalous trajectories. The trajectories corresponding to the top three distances are displayed in Figure 7b, and the trajectories displaying the corresponding maximum distance difference are shown in Figure 7d. The visual displays of all the distances and the differences in Figure 7 are further conductive to find the abnormal trajectories. Therefore the distance judgment method and the differences judgment method are chosen in this paper.

**Figure 8:**
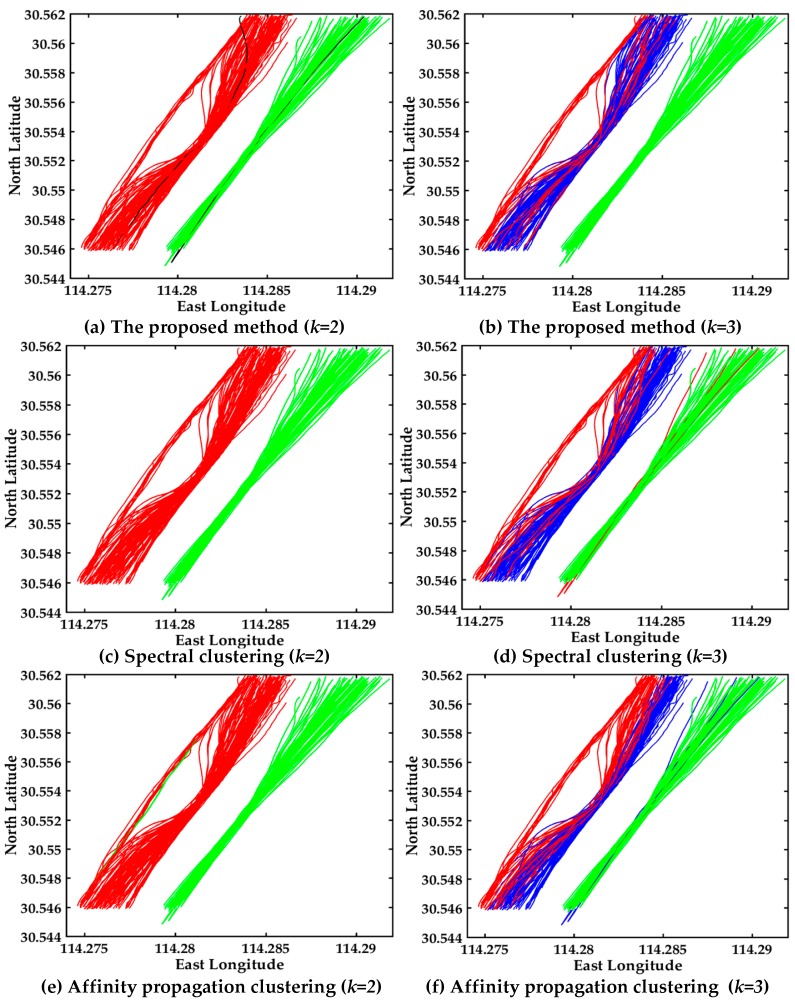
Visual display of different algorithms. (**a**) Multi-step clustering algorithm (*k = 2*); (**b**) Multi-step clustering algorithm (*k = 3*); (**c**) Spectral clustering (*k = 2*); (**d**) Spectral clustering (*k = 3*); (**e**) Affinity propagation clustering (*k = 2*); (**f**) Affinity propagation clustering (*k = 3*).

The visual display of AIS trajectories of 161 vessels in the bridge area are shown in Figure 8, where there are three bridge openings and two bridge piers in the visual display. The vessel trajectories converge before entering the bridge opening and after leaving the bridge opening in the left trajectories. The proposed multi-step clustering algorithm is compared with the spectral clustering and affinity propagation clustering, the experimental comparison results of three clustering algorithms are shown in Figure 8.

The clustering result with multi-step clustering algorithm when *k = 2* is shown in Figure 8a. The trajectories are divided into two classes, where red and green represent different classes, and black trajectories are the cluster centers. Certainly there is no intersection of clustering results, therefore the performance is good. The clustering result with spectral clustering when *k = 2* is shown in Figure 8c. The result is the same with multi-step clustering algorithm, however the result doesn’t show the cluster centers. The clustering performance of the affinity propagation when *k = 2* is shown in Figure 8e, and the misclassification trajectories are green in the red trajectories. As can be seen in Figure 8a,c,e, the red and green trajectories denote the different classes. The accuracy of the multi-step clustering algorithm and spectral clustering are both 100%, while the affinity propagation clustering has a green trajectory in red trajectories. The multi-step clustering algorithm has the better clustering performance when clustering center *k = 2*, see Figure 8a,c,e.

In order to further verify the clustering number accuracy and the effectiveness of the proposed algorithm, we compared the clustering result of three algorithms when *k = 3*. The visual displays of the multi-step clustering algorithm, spectral clustering and affinity propagation when *k = 3* are shown in Figure 8b,d,f. The red, blue and green trajectories represent different classes, respectively. As can be seen clearly in Figure 8b,d,f, the multi-step clustering algorithm has better clustering performance than the other two algorithms when *k = 3*. There is no other trajectory in the green trajectories in Figure 8b. The multi-step algorithm of fusion with DTW, PCA and the improved center clustering algorithm can make up for the shortcomings of traditional clustering algorithms, and also promote the performance of the clustering results.

In conclusion, it’s obvious that the performance of the proposed multi-step clustering algorithm is better than that of the other two algorithms. The experimental results verify the effectiveness of the multi-step method. Certainly the vessel traffic flow pattern and customary routes can be easily found in Figure 8. The two classes of vessels trajectories in the bridge area waters are clearly seen from the AIS trajectory clustering visualization.

### 4.3. Clustering Analysis of AIS Trajectory in the Mississippi River

#### 4.3.1. Visualization of the Distance Matrix 

The Mississippi River is the longest river in the United States, and it is also the World’s fourth longest river. The Mississippi River has convenient and cheap shipping resources, abundant mineral resources and unique agricultural resources. Therefore, the complex traffic flow characteristics and changeable environment condition have made the Mississippi River a focus of research. The experiment dataset was collected in Mississippi River and includes 106 AIS trajectories, or 2442 points altogether.

Data cleansing is the first step of trajectory visualization, then the 67 trajectories (altogether 1532 coordinate points) of different vessels are preserved after data cleansing. The traffic flows are complex, and the trajectories are rather messy. In order to clearly shown the performance of the proposed method, the trajectories are divided into two classes according to the ship course. There are 37 trajectories of up-bound vessels and 30 trajectories of down-bound vessels, and certainly they have the opposite course. In order to clearly mine the traffic flow characteristics and patterns, the experiments are carried out on the trajectories of 37 up-bound vessels and 30 down-bound vessels, respectively. The 37 trajectories consisted of 744 coordinate points, and the 30 trajectories consisted of 788 coordinate points.

**Figure 9:**
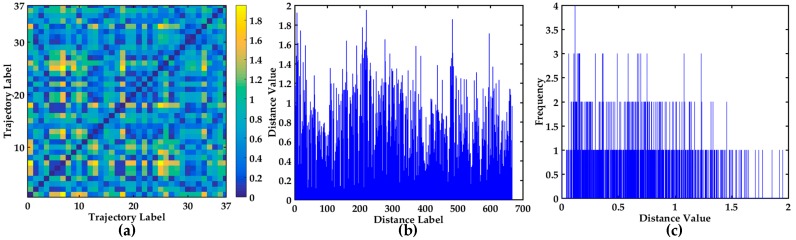
Visualization of 37 × 37 distance matrix. (**a**) 2D image visualization of distance matrix; (**b**) The bar chart of all the different values in 37 × 37 distance matrix; (**c**) The statistical histogram of all the non-repetitive distances in 37 × 37 distance matrix.

The similarity measurement method DTW is implemented to calculate the distance, then the 37 × 37 dimensional matrix of up-bound vessels and the 30 × 30 dimensional matrix of down-bound vessels are calculated, respectively. The visual display of the 37 × 37 distance matrix is shown in Figure 9. As seen in Figure 9a, and 2D image visualization of distance matrix can clearly show the symmetry of the distance matrix. The different colors express different values. The bar chart display of all the non-repetitive distances in 37 × 37 distance matrix is shown in Figure 9b, which is helpful to observe outliers. The trajectory is relatively smooth and regular from Figure 9b. The statistical histogram of all the non-repetitive distances is shown in Figure 9c, which is conductive to finding the distribution of all the distances. The range of the distances is [0, 2], and further indicates that the trajectories are relatively smooth.

**Figure 10:**
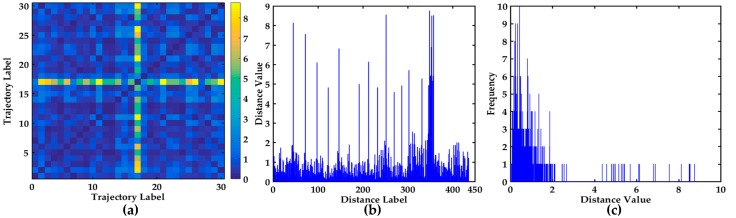
Visualization of 30 × 30 distance matrix. (**a**) 2D image visualization of distance matrix; (**b**) The bar chart of all the different values in 30 × 30 distance matrix; (**c**) The statistical histogram of all the non-repetitive distances in 30 × 30 distance matrix.

The different visual display of the distance matrix of the 30 down-bound trajectories in the Mississippi River are shown in Figure 10, which is helpful to further observe the distances and analyze the abnormal trajectories. The 2D image visualization can clearly show the symmetry of the distance matrix in Figure 10a. As shown in Figure 10b, the X-axis represents the distance label, and the Y-axis represents the distance value. The Y-axis range is [0, 9], and the trajectory is relatively complex and irregular from Figure 10b. The bar chart is conducive to distinguishing abnormal trajectories. As shown in Figure 10c, the statistical histogram of all the non-repetitive distances is conductive to finding the distribution of all the distances. The range of the distances is mainly [0, 2], however, there are many large values. The statistical histogram can further indicate that the trajectories are relatively irregular.

#### 4.3.2. Visualization of the Clustering Number

The top 10 eigenvalues of the distance matrix and the corresponding accumulative contribution rate are listed in Table 3. It’s clear that the accumulative contribution rate of the top three eigenvalues is 96.58% which is more than 95%, so the number of cluster is set to be *k = 3* in our multi-step clustering algorithm. When the 37 trajectories are clustered, *k = 3* is taken as the number of clusters in the improved center clustering algorithm. Certainly the experiment will compare the clustering performance when *k = 2* and *k = 3* to prove the effectiveness and feasibility of the proposed multi-step algorithm.

The top 10 EV and ACR are listed in Table 4. It’s clear that the accumulative contribution rate of the top three eigenvalues is 97.57% that is more than 95%, so the number of clusters is set to be *k = 3* in our multi-step clustering algorithm. When the 30 trajectories are clustered, *k = 3* is taken as the number of clusters in the improved center clustering algorithm. To further prove the effectiveness and feasibility of the proposed multi-step algorithm, the experiment will compare the clustering performance when *k = 2* and *k = 3*.

#### 4.3.3. Visualization of Clustering Results about 37 Up-Bound Vessels in the Mississippi River

The number of the clusters is set to be three according to PCA, and the centers of the cluster are selected by the improved center selection algorithm. The distances between all the trajectories are sorted in descending order. There are $C_{37}^{2}=666$ distances between 37 trajectories, $d_{i},\;i=1\;,\ldots ,\;666$ represents the *i^th^* distance value. $\xi_{i}$ denotes threshold value, and $\xi_{i}=|d_{i}-d_{i+1}|\;,\;i=1\;,\ldots ,\;666$ is the absolute value of difference between two adjacent distances. The average value of the distances between the trajectories is set to the threshold value $\xi =(\sum_{i=1}^{666}d_{i})/666$. If $\xi_{i}>\xi$ expresses the corresponding trajectories may be abnormal trajectory, then further confirm whether they are abnormal trajectories according course over ground (COG) and speed over ground (SOG).

The top 10 distances and the difference $\xi_{i}$ between two adjacent distances are listed in Table 5. The threshold value is $\xi =(\sum_{i=1}^{666}d_{i})/666=0.6602$, and the minimum distance is $d_{\min}=0.0414$. It can be seen clearly that the maximum distance is $d_{\max}=1.951599$, and the maximum difference $\xi_{i}$ is 0.08325. The values $\xi_{i}$ in Table 5 are all smaller than the threshold value.

**Figure 11:**
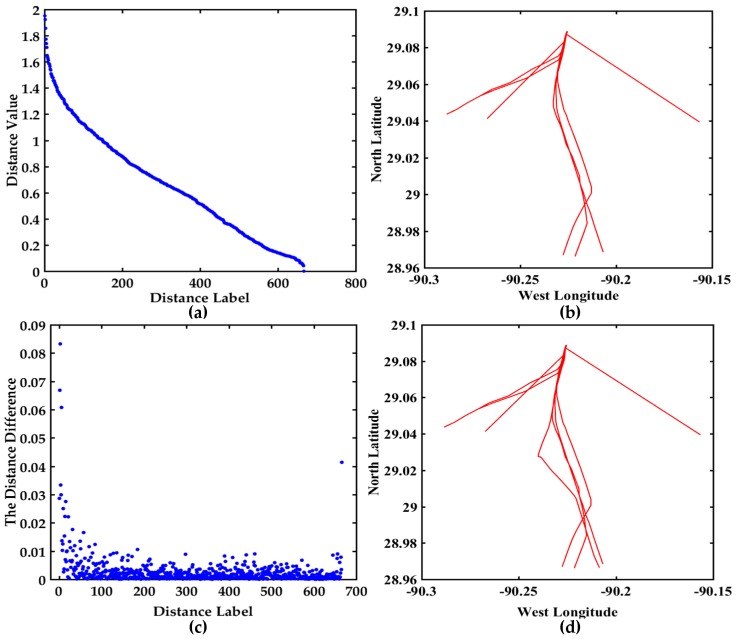
Visualization of the distance values and the distance differences between 37 trajectories, (**a**) The descending order map of distance values; (**b**) The trajectories display corresponding to the top 6 distances; (**c**) The distance difference display between two adjacent distance values in (a); (**d**) The trajectories display corresponding to the top three distance differences.

In order to further see the variation trend of all the distances and find the hidden abnormal trajectories, all the distances values and the distance differences among the 37 trajectories are displayed in Figure 11. The descending order map of all the distances is shown in Figure 11a. The Y-axis range is [0, 2], and the variation trend of the distances is slow. The trajectories corresponding to the top six distances are shown in Figure 11b, and they may be abnormal trajectories. The distance difference between two adjacent distance values is shown in Figure 11c, where the maximum value of the Y-axis is 0.083250 and is less than 0.6602.

In order to further see the variation trend of all the distances and find the hidden abnormal trajectories, all the distances values and the distance differences between 37 trajectories are displayed in Figure 11. The descending order map of all the distances are shown in Figure 11a. The range of Y-axis is [0, 2], and the variation trend of the distances is slow. The trajectories corresponding to the top 6 distances are shown in Figure 11b, and they may be abnormal trajectories. The distance difference between two adjacent distance values is shown in Figure 11c, the maximum value of Y-axis is 0.083250 and is less than 0.6602. It can be seen from Figure 11a that the curve is relatively smooth. The trajectories corresponding to the top three distance differences are displayed in Figure 11d. The trajectories in Figure 11b,d are further confirmed whether they are abnormal trajectories based on COG and SOG. The COG and SOG don’t satisfy the abnormal condition, then the trajectories in Figure 11b,d are not the anomaly trajectories. The visualization of distance and the distance difference can further contribute to find the hidden anomalous trajectories.

**Figure 12:**
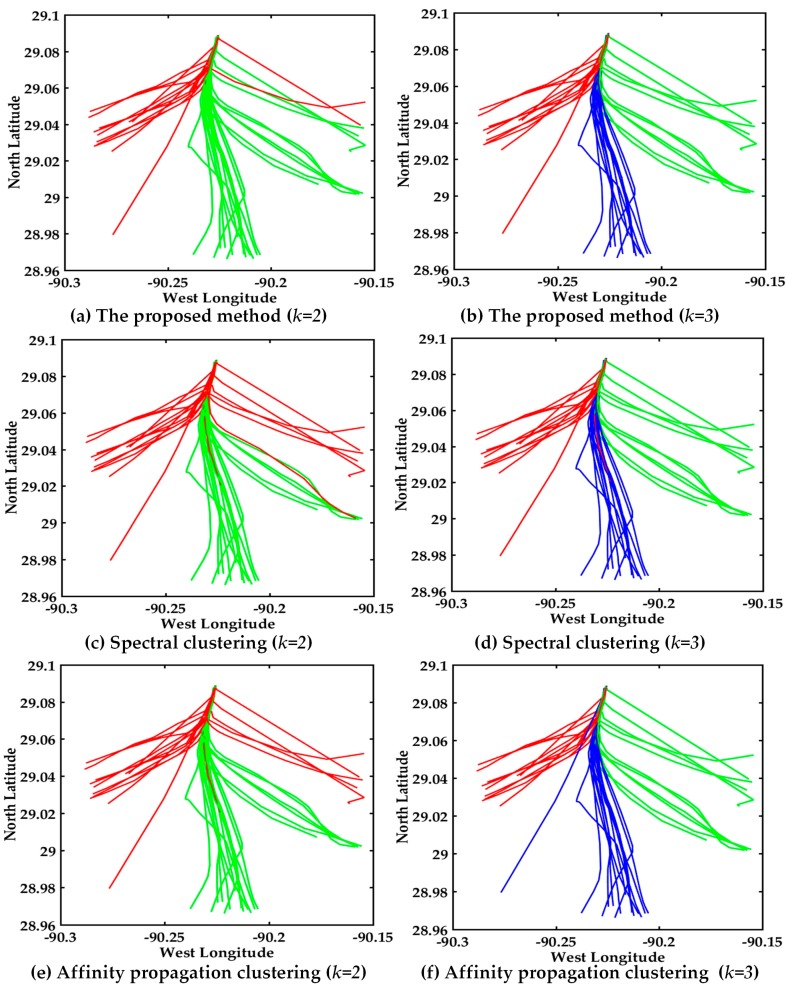
Visual display of different algorithms about 37 trajectories, (**a**) Multi-step clustering algorithm (*k* = 2); (**b**) Multi-step clustering algorithm (*k* = 3); (**c**) Spectral clustering (*k* = 2); (**d**) Spectral clustering (*k* = 3); (**e**) Affinity propagation clustering (*k* = 2); (**f**) Affinity propagation clustering (*k* = 3).

The dataset is collected from different vessels at the exit of Mississippi River Delta, where there are many routes. A visual display of AIS trajectories in the Mississippi River using three algorithms is shown in Figure 12, the experimental comparison results of the three clustering algorithms further illustrates the effectiveness of the proposed multi-step clustering algorithm.

The clustering results with multi-step clustering algorithm, spectral clustering and affinity propagation clustering when *k = 2* are shown in Figure 12a,c,e. The trajectories are clustered into two classes, where red and green represent different classes, respectively. It’s clear that the clustering performance of the proposed multi-step algorithm is better than the other two algorithms when *k = 2*.

The performance of *k = 3* are shown in Figure 12b,d,f. The trajectories are clustered into three classes, where red, green and blue represent different classes, respectively. As shown in Figure 12d, the misclassified trajectory appears as a red line in the blue trajectories. The misclassified trajectory is the blue line in the red trajectories in Figure 12f. The experiment further verified the validity of the proposed method. The three clusters are clearly seen from Figure 12b, and the customary routes in the Mississippi River Delta exit can be easily found. The trajectories of the 37 up-bound vessels are relatively smooth and regular.

#### 4.3.4. Visualization of Clustering Results of 30 Down-Bound Vessels in the Mississippi River

The number of clusters in 30 trajectories is set to be three according to PCA, and the centers of the cluster are selected by the improved center selection algorithm. The distances between all the trajectories are sorted in descending order. There are $C_{30}^{2}=435$ distances between 30 trajectories, $d_{i},\;i=1\;,\ldots ,\;435$ represents the *i^th^* distance value. $\xi_{i}$ denotes threshold value, and $\xi_{i}=|d_{i}-d_{i+1}|\;,\;i=1\;,\ldots ,\;435$ is the absolute value of difference between two adjacent distances. The average value of the distances between the trajectories is set to the threshold value $\xi =(\sum_{i=1}^{435}d_{i})/435$. If $\xi_{i}>\xi$ it expresses that the corresponding trajectories may be abnormal trajectories, and then further confirm whether they are abnormal trajectories according to course over ground (COG) and speed over ground (SOG).

The traffic flow in the Mississippi River Delta area is busy and complex, and sea routes are also changeable. Then the distance difference between the trajectories will be relatively large. In this experiment, the traffic flow in Mississippi River Delta exit is so complex that there are many routes in different directions at the river exit. The top 10 distances and the differences $\xi_{i}$ between two adjacent distances are listed in Table 6. The threshold value is $\xi =(\sum_{i=1}^{435}d_{i})/435=1.014$, and the minimum distance is $d_{\min}=0.0594$. It is obvious that the maximum distance is $d_{\max}=8.76072$, and the maximum difference $\xi_{i}$ is 0.678266. The values $\xi_{i}$ in Table 6 are all smaller than the threshold value. The difference $\xi_{i}$ in Table 6 may not be the maximum difference between the trajectories, therefore all the distances and the distance differences are visualized to further find the abnormal trajectory.

**Figure 13:**
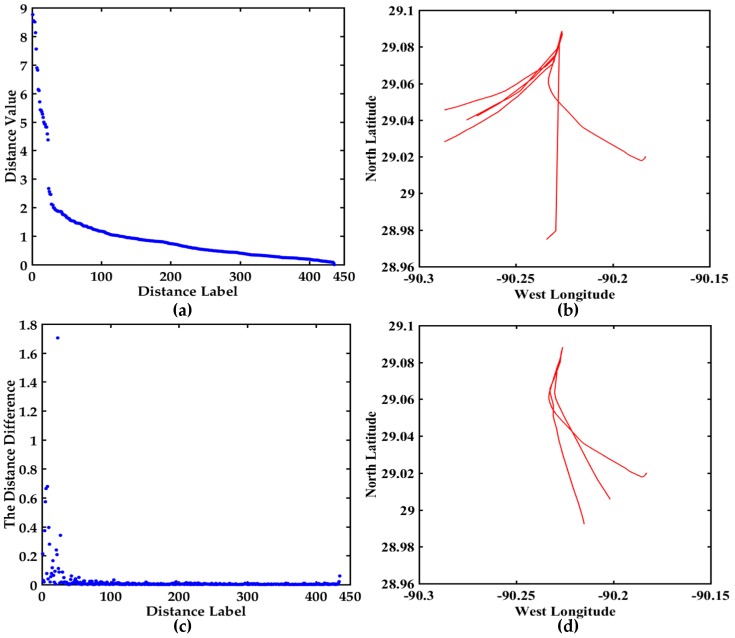
Visualization of the distance values and the distance differences between 30 trajectories, (**a**) The descending order map of distance values; (**b**) The trajectories display corresponding to the top 6 distances; (**c**) The distance difference display between two adjacent distance values in (**a**); (**d**) The trajectories display corresponding to the maximum distance difference.

All the distances and the distance differences are shown in Figure 13. The descending order map of all the distances is shown in Figure 13a. The Y-axis range is [0, 9] and the variation trend is obvious. The trajectories corresponding to the top six distances are shown in Figure 13b, and they may be abnormal trajectories. The distance difference between two adjacent distance values is shown in Figure 13c, the maximum value of the Y-axis is 1.7040, which is further helpful to find the hidden anomalous trajectories. The trajectories corresponding to the maximum distance difference are displayed in Figure 13d. The trajectories in Figure 13b,d are further confirmed whether they are abnormal trajectories based on COG and SOG. For $\max\xi_{i}>\xi$ from Table 6 and Figure 13, the trajectories may be anomalous. However the COG and SOG don’t satisfy the abnormal condition, so the corresponding trajectories are not anomalous trajectories. The maximum distance difference between the 23rd distance and the 24th distance denotes the trajectories are relatively irregular.

**Figure 14:**
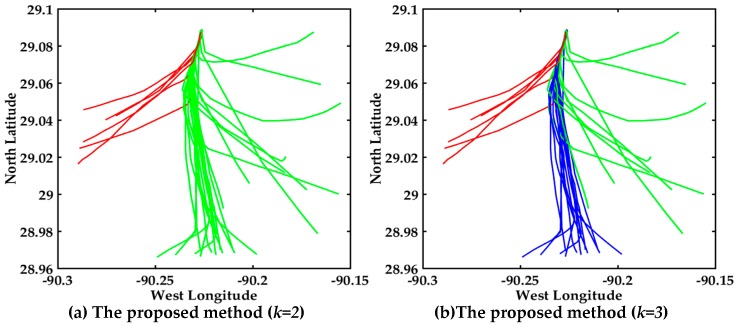

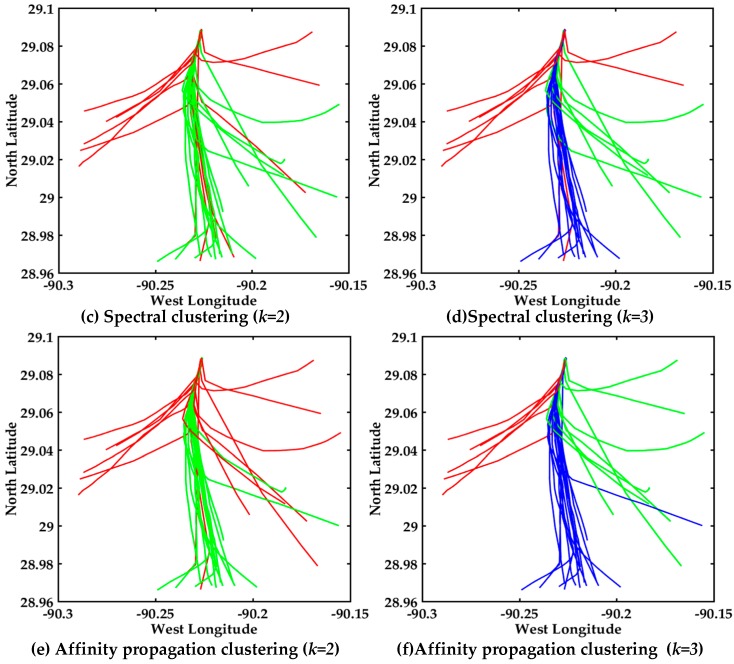
Visual display of different algorithms about 30 trajectories, (**a**) Multi-step clustering algorithm (*k* = 2); (**b**) Multi-step clustering algorithm (*k* = 3); (**c**) Spectral clustering (*k* = 2); (**d**) Spectral clustering (*k* = 3); (**e**) Affinity propagation clustering (*k* = 2); (**f**) Affinity propagation clustering (*k* = 3).

The heavy and complex traffic flow in Mississippi River Delta leads to trajectories that are irregulary and unsmooth compared with trajectories in inland waters, and thus it is not conducive to clustering. Visual display of the AIS trajectories of three algorithms in the Mississippi River is shown in Figure 14, where the experimental comparison results of three clustering algorithms further illustrate the effectiveness of the proposed multi-step clustering algorithm.

The trajectories in the Mississippi River Delta are more irregular than the trajectories in inland waters from Figure 8 and Figure 14. The clustering result with the multi-step clustering algorithm, spectral clustering and affinity propagation clustering when *k = 2* are shown in Figure 14a,c,e. The trajectories are clustered into two classes, where red and green represent different classes, respectively. It’s very clear that the clustering performance of the proposed multi-step algorithm is better than that of the other two algorithms when *k = 2*.

The performance of *k = 3* is shown in Figure 14b,d,f. The trajectories are automatically clustered into three classes, where red, green and blue represent different classes, respectively. As shown in Figure 14d, the misclassified trajectories are the red lines in the blue and green trajectories. As shown in Figure 14f, the misclassified trajectory is the green line in the blue trajectories. The three clusters are clearly seen from Figure 14b, where the customary routes in the Mississippi River Delta exit can be easily found. It can be seen that the trajectories in Figure 12 are more regular and smoother than those in Figure 14. Obviously, the overall clustering performance of the proposed algorithm is also suitable for dealing with irregular trajectories and more prone to cluster trajectories.

### 4.4. Time Complexity Analysis

The time complexity of the proposed multi-step algorithm includes the calculation of the DTW, PCA and the improved center clustering algorithm, the time complexity of DTW and PCA are both $O(n^{2})$, the time complexity of the improved center clustering algorithm is O(k·(n−k)), the time complexity of spectral clustering is $O(n^{2})$, the time complexity of affinity propagation clustering is $O(n^{2}\cdot \log(n))$, where *n* represents the number of AIS trajectories, *k* is the number of cluster centers. The time and accuracy comparison results of different algorithms are listed in the Table 7.

As shown in Table 7, the running time of DTW in bridge area waterway is 495.599 s. The dataset in bridge area waterway has 161 trajectories (altogether 25,678 coordinate points), so the running time of DTW is relatively longer. The clustering time of the proposed method in bridge area waterway is 1.834 s, the spectral clustering is 2.598 s, and the affinity propagation clustering 3.517 s. The proposed method saves 0.764 s and 1.683 s respectively, compared with the spectral clustering and the affinity propagation clustering. The clustering accuracies of the three algorithms in the bridge area waterway are 100%, 100%, and 99.5%. The time complexity and the running time of the multi-step clustering algorithm are both less than the other two algorithms, moreover the clustering accuracy in the bridge area waterway is better.

It can be observed that the trajectories of the 37 up-bound vessels consisted of 744 points and the running time of DTW is 5.89 s. The clustering times of the three algorithms in the Mississippi River are 0.836 s, 1.606 s, and 1.194 s, respectively. The clustering accuracies of the three algorithms in the Mississippi River are 100%, 97.3%, and 97.3%. The time complexity and the running time of the multi-step clustering algorithm are both less than those of the other two algorithms, moreover the clustering accuracy in the bridge area waterway is better than that of the other two algorithms.

All the trajectories of the 30 down-bound vessels have 788 points. The running time of DTW is 2.075 s. The clustering time of the three algorithms in the Mississippi River are 0.737 s, 1.139 s, and 1.235 s, respectively. The clustering accuracy of the three algorithms in the Mississippi River are 96.67%, 86.67%, and 96.67%. The clustering accuracies of the three algorithms in the bridge waters are 100%, 100%, and 99.5%. Compared with the spectral clustering, the clustering time of the proposed clustering algorithm is 0.402 s shorter and the clustering accuracy improves by 10%.

The time complexity and the running time of the multi-step clustering algorithm are both less than those of the other two algorithms, moreover the clustering accuracy in the bridge area waterway (*k = 2*) and the clustering accuracy in the Mississippi River (*k = 3*) are greater than the other two algorithms, respectively. The proposed multi-step clustering algorithm has higher clustering accuracy and lower time complexity both in the bridge area waterway and in the Mississippi River from the Table 7. The time complexity analysis further demonstrates the effectiveness and clustering accuracy of the proposed multi-step algorithm.

### 4.5. Discussion

The multi-step algorithm fuses DTW, PCA and the improved center clustering algorithm, which is very suitable for clustering AIS trajectories. The experiments have confirmed the validity and the feasibility of the proposed method. DTW can effectively calculate the distance between trajectories, and the improved center clustering algorithm has higher accuracy and lower time complexity than spectral clustering and affinity propagation clustering. The experiment results have demonstrated a huge potential of the proposed multi-step method for trajectories clustering. Moreover, the traffic pattern and customary routes can be found from the clustering results. Fusion with PCA and the improved center clustering algorithm are the innovative points of this paper. It can automatically select cluster centers based on the distance between trajectories, and simultaneously improved the clustering accuracy with lower time complexity.

## 5. Conclusions

In this paper, a new trajectory clustering method (termed the multi-step clustering method) was proposed to find the customary vessel routes and detect abnormal trajectories. It effectively integrated both PCA and the improved center selection algorithm into a unified mathematical framework. The trajectory clustering problem was effectively solved by the multi-step clustering method. Numerous experiments have been conducted on two realistic datasets of a bridge area waterway and the Mississippi River to compare the proposed method with the other two state-of-the-art clustering methods. The experimental results have demonstrated the superior performance of the proposed method in terms of both quantitative and qualitative analysis. Therefore, the proposed multi-step clustering method has higher accuracy and lower time complexity than the spectral clustering and affinity propagation clustering with a specified cluster number. However, the improved center selection algorithm must be selected according to the distance between different trajectories. Thus, the next step in our future work is to study in depth the automatic method for selecting the cluster centers based on the navigation direction. On the other hand, the automatic threshold selection is also a next research focus.

## Figures and Tables

**Table 1:** The top 10 eigenvalues (EV) and the corresponding accumulative contribution rate (ACR) with PCA.

**EV**	114.732	40.9095	2.94698	1.30154	0.42465	0.20297	0.17504	0.09430	0.05381	0.03565
**ACR**	71.26%	96.67%	98.50%	99.31%	99.57%	99.70%	99.81%	99.87%	99.90%	99.92%

**Table 2:** The top 10 distances (TD) and the difference $\xi_{i}$ between two adjacent distances.

**TD**	4.1610	3.9106	3.7622	3.1241	2.9842	2.9264	2.9071	2.8873	2.8560	2.8220
$\xi_{i}$	0.2504	0.1484	0.6381	0.1399	0.0578	0.0193	0.0198	0.0313	0.0340	0.0051

**Table 3:** The top 10 EV and the corresponding ACR with PCA about 37 trajectories.

**EV**	20.4118	9.639	5.68566	0.61237	0.21397	0.12671	0.07561	0.06494	0.04029	0.02758
**ACR**	55.17%	81.22%	96.58%	98.24%	98.82%	99.16%	99.36%	99.54%	99.65%	99.72%

**Table 4:** The top 10 EV and the corresponding ACR with PCA about 30 trajectories.

**EV**	22.2254	3.88804	3.1564	0.27695	0.23190	0.08530	0.04390	0.03991	0.01813	0.00976
**ACR**	74.08%	87.04%	97.57%	98.49%	99.26%	99.55%	99.69%	99.83%	99.88%	99.92%

**Table 5:** The top 10 TD and the difference $\xi_{i}$ between two adjacent distances.

**TD**	1.9516	1.9230	1.8561	1.7729	1.7395	1.7096	1.6488	1.6352	1.6250	1.6124
$\xi_{i}$	0.0286	0.0669	0.0832	0.0334	0.0299	0.0608	0.0136	0.0102	0.0126	0.0250

**Table 6:** The top 10 distances (TD) and the difference $\xi_{i}$ between two adjacent distances.

**TD**	8.7607	8.5478	8.5184	8.5003	8.1277	7.5552	6.8915	6.8151	6.1368	6.0978
$\xi_{i}$	0.2129	0.0294	0.0181	0.3726	0.5725	0.6637	0.0764	0.6783	0.0390	0.3947

**Table 7:** The time and accuracy comparison results of different algorithms.

	Multi-Step Clustering Algorithm	Spectral Clustering	Affinity Propagation Clustering
Time complexity	$O(n^{2})$	$O(n^{2})$	$O(n^{2}\cdot \log(n))$
*T_D-B_*	495.599s	495.599s	495.599s
*T_B_*	1.834s	2.598s	3.517s
*A_B-2_*	100%	100%	99.5%
*T_D-M37_*	5.89s	5.89s	5.89s
*T_M37_*	0.836s	1.606s	1.194s
*A_M37-3_*	100%	97.3%	97.3%
*T_D-M30_*	2.075s	2.075s	2.075s
*T_M30_*	0.737s	1.139s	1.235s
*A_M30-3_*	96.67%	86.67%	96.67%

*T_D-B_* means the running time of DTW in the bridge area waterway, *T_B_* denotes the clustering time in the bridge area waterway, *A_B-2_* expresses the clustering accuracy in the bridge area waterway (*k = 2*), *T_D-M37_*, means the running time of DTW of 37 trajectories in the Mississippi River, *T_M37_* denotes the clustering time of the 37 trajectories in the Mississippi River, *A_M37-3_* express the clustering accuracy of the 37 trajectories (*k* = 3) in the Mississippi River, *T_D-M30_*, means the running time of DTW about 30 trajectories in the Mississippi River, *T_M30_* denotes the clustering time of the 30 trajectories in the Mississippi River, *A_M30-3_* express the clustering accuracy of 30 trajectories (*k* = 3) in the Mississippi River.
